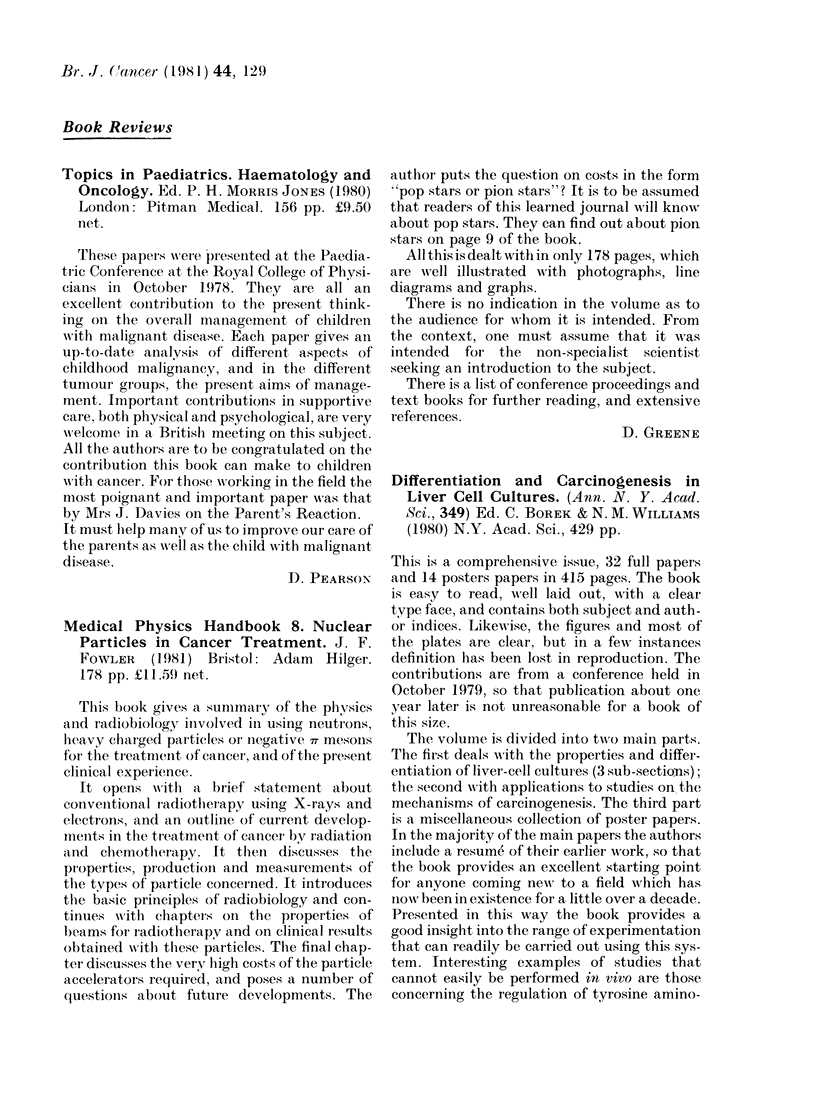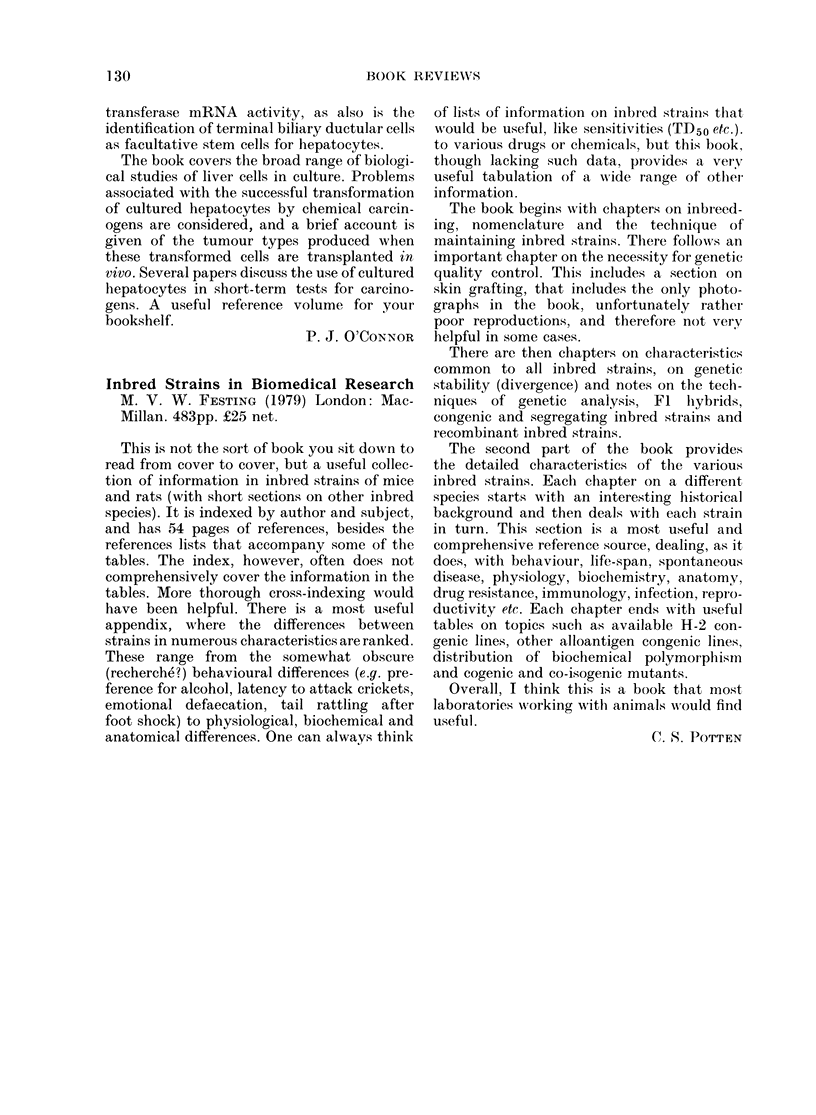# Differentiation and Carcinogenesis in Liver Cell Cultures

**Published:** 1981-07

**Authors:** P. J. O'Connor


**Differentiation and Carcinogenesis in Liver Cell Cultures.** (*Ann. N. Y. Acad. Sci.*, **349**) Ed. C. Borek & N. M. Williams (1980) N.Y. Acad. Sci., 429 pp.

This is a comprehensive issue, 32 full papers and 14 posters papers in 415 pages. The book is easy to read, well laid out, with a clear type face, and contains both subject and author indices. Likewise, the figures and most of the plates are clear, but in a few instances definition has been lost in reproduction. The contributions are from a conference held in October 1979, so that publication about one year later is not unreasonable for a book of this size.

The volume is divided into two main parts. The first deals with the properties and differentiation of liver-cell cultures (3 sub-sections); the second with applications to studies on the mechanisms of carcinogenesis. The third part is a miscellaneous collection of poster papers. In the majority of the main papers the authors include a resumé of their earlier work, so that the book provides an excellent starting point for anyone coming new to a field which has now been in existence for a little over a decade. Presented in this way the book provides a good insight into the range of experimentation that can readily be carried out using this system. Interesting examples of studies that cannot easily be performed *in vivo* are those concerning the regulation of tyrosine aminotransferase mRNA activity, as also is the identification of terminal biliary ductular cells as facultative stem cells for hepatocytes.

The book covers the broad range of biological studies of liver cells in culture. Problems associated with the successful transformation of cultured hepatocytes by chemical carcinogens are considered, and a brief account is given of the tumour types produced when these transformed cells are transplanted *in vivo*. Several papers discuss the use of cultured hepatocytes in short-term tests for carcinogens. A useful reference volume for your bookshelf.

P. J. O'Connor